# Quality of life after stroke: impact of clinical and sociodemographic factors

**DOI:** 10.6061/clinics/2017/e418

**Published:** 2018-09-24

**Authors:** Maria José Melo Ramos-Lima, Ismênia de Carvalho Brasileiro, Tamires Layane de Lima, Pedro Braga-Neto

**Affiliations:** I Centro de Ciencias da Saude, Universidade Estadual do Ceara, Fortaleza, CE, BR; II Departamento de Fisioterapia, Universidade Estacio do Ceara, Fortaleza, CE, BR; III Divisao de Neurologia, Departamento de Medicina Clinica, Universidade Federal do Ceara, Fortaleza, CE, BR

**Keywords:** Stroke, Quality of life, Disability

## Abstract

**OBJECTIVES:**

The aim of the study was to analyze the impact of ischemic stroke on health-related quality of life (QoL) and associate this event with individuals' clinical and sociodemographic characteristics.

**METHODS:**

We investigated the clinical and demographic aspects of stroke patients. The Modified Rankin Scale, National Institutes of Health Stroke Scale (NIHSS) and the Stroke Specific Quality of Life Scale (SS-QoL) were used for correlation analysis.

**RESULTS:**

Among 131 patients with ischemic stroke, 53.4% of patients presented with moderate to severe disability on the Rankin Scale. According to the SS-QoL, several QoL domains were compromised. QoL was significantly negatively correlated with the values of the Rankin and NIHSS scales, indicating lower QoL among people with worse functional status and greater clinical severity of stroke (*p*<0.001). The use of orthosis and total anterior circulation infarct subtype of stroke led to a more marked reduction in QoL.

**CONCLUSION:**

The present study described an inversely proportional relationship between the severity of stroke, disability and QoL. The use of orthosis also had a negative impact on QoL. Early identification of these factors could promote better interventions for individuals with ischemic stroke, minimizing disabilities and improving QoL.

## INTRODUCTION

Stroke is one of the main causes of death and functional inability worldwide 1. In the last decade, the global incidence of stroke has increased by 20% in low- and middle-income countries such as Brazil 2. Stroke is the third most common cause of death in developed countries and the main cause of death in Latin America. Brazil stands out as having the highest rate of mortality from stroke in the Americas 3,4. In a recent study, the overall survival rate was 47% 5.

The high prevalence of stroke in the Brazilian population is a major economic and social burden 6. The physical, social and psychological consequences arising from this condition are devastating—approximately 90% of survivors have some type of disability 7. Stroke has a direct impact on health systems, resulting in high costs, and is also considered a global public health problem due to serious disabilities, functional limitations and compromised quality of life (QoL) 8.

The impact of stroke on people's lives represents an important challenge for society. In addition to being a sudden event, stroke affects both the individual and family, which is overall unprepared to deal with the process of rehabilitation or the disabilities that result from this condition. As a result, a high number of people are unable to work and receive financial assistance after stroke 9,10.

In Brazil, few studies on QoL after stroke have been developed. The need to understand the extent, magnitude of the effects of stroke and the important implications for health is evident. How changes over time in physical, emotional, cognitive and community participation influence QoL is necessary to delineate areas 11. In addition, there is a need to establish a basis for developing future care models for stroke survivors by improving their QoL.

Stroke survivors face a new challenge, i.e., living with disabilities. Patients with physical and/or mental sequelae require specific rehabilitation to achieve functional recovery. Moreover, family, community and social reintegration, as well as maintenance of recovery level are of paramount importance for achieving good QoL 11.

Studies on QoL are important for assessing the impact of an individual's life on society. Studies undertaken with stroke patients have demonstrated that stroke affects various domains of quality of life, compromising functionality 7,9,12. However, there are few studies focusing on this topic in Latin America. It is of fundamental importance to elaborate specific programs for functional recovery and develop health policies that aim to achieve social inclusion and readaptation to work, as well as health promotion strategies aiming to control risk factors for stroke patients. The aim of this study was to analyze the impact of ischemic stroke (IS) on health-related QoL and associate this event with individuals' clinical and sociodemographic characteristics.

## METHODS

### Sample and subjects

We conducted a cross-sectional observational study undertaken between February and November 2016 in 2 stroke care outpatient centers that specialize in caring for patients with stroke in the Northeast region of Brazil. These 2 centers are the reference for public care in stroke in the city of Fortaleza. IS was diagnosed in the emergency room based on neurological examination and neuroimaging studies. The inclusion criteria were as follows: 18 years of age or older, one or more diagnoses of IS, and stroke event between six and 12 months. The exclusion criteria were as follows: deafness; associated neurological illness such as Alzheimer's disease, Parkinson's disease or other degenerative disease; language-related limitations that prevented responses to evaluation; and cognitive deficits. Sixty-seven patients were excluded; twenty-two were excluded due to aphasia or dysarthria, twenty-six due to neurological diseases, four due to an important auditory deficit and fifteen due to cognitive deficits. The final sample comprised 131 patients. The Local Research Ethics Committee of the institutions approved the study in accordance with the Helsinki Declaration of 1975, which was revised in 1983. All subjects provided informed consent.

### Clinical evaluation

We collected the following sociodemographic and clinical data: age, gender, marital situation, skin color, profession, actual job market and eventual job change after stroke, financial assistance after stroke, number of people residing with the patient, income, level of education, number of stroke events, affected hemisphere, Bamford classification, thrombolytic therapy, time post stroke, duration of hospitalization and time between stroke ictus and first treatment.

We also obtained a detailed clinical history and investigated medical records regarding the occurrence of hypertension, sedentarism, smoking, drinking, dyslipidemia, diabetes mellitus, previous stroke, cancer, obesity, cardiac insufficiency, coronary disease, acute myocardial infarction, transient ischemic attack, cardiomyopathy, chronic obstructive pulmonary disease, kidney failure, peripheral artery disease and drug abuse. These diseases were diagnosed based on international criteria, medical records and medical consultation.

The Mini-Mental State Examination (MMSE) was used for cognitive screening 13. The Modified Rankin Scale (mRS) was used to assess stroke disability and the National Institutes of Health Stroke Scale (NIHSS) was collected on admission to determine stroke severity. For the mRS, the score was divided between 0 to 2 (mild disability) and 3 to 5 (moderate to severe disability). For the NIHSS, a score of up to 4 points was considered a minor stroke, 5 to 15 points a moderate stroke, 16 to 20 points a severe stroke, and 21 points or over a very severe stroke. All participants were further classified according to Trial of Org 10 172 in Acute Stroke Treatment (TOAST) criteria as follows: total anterior circulation stroke syndrome (TACS), partial anterior circulation stroke syndrome (PACS), posterior circulation stroke syndrome (POCS) and lacunar stroke syndrome (LACS) 14.

To evaluate QoL, we applied the Stroke Specific Quality of Life Scale (SS-QoL). The SS-QoL is an instrument specifically used to assess health-related QoL among individuals who experienced stroke. The SS-QoL has been validated and transculturally adapted for Brazil 15; it has 49 items in 12 domains, varying from 49 to 245 points, with responses varying from 1 to 5 points. Higher values indicate better health-related QoL. One study on this instrument undertaken in Germany defined scores less than 60% (<147 points) as constituting low QoL, and the same criteria were used in the present study 16. All questionnaires were performed face-to-face with the patient in the medical consultation obeying the specific guidelines in the application of each one.

### Statistical analysis

Descriptive and inferential statistics were used with Statistical Package for the Social Sciences software, version 21.0 and R software, version 3.3.1. For categorical variables, absolute frequencies and percentages were determined, while for numerical variables, the means and standard deviation were established. SS-QoL values were compared with nominal social and clinical characteristics using the Mann-Whitney test to compare variables with two categories or the Kruskal-Wallis test when the variable in question encompassed more than two categories. Dunn's test was applied for post hoc comparisons of the Kruskal-Wallis test when relevant, and superscript letters above the categories were used to highlight statistically significant differences between the multiple comparisons.

A multiple linear regression model was adjusted to establish the extent of the influence of multiple clinical and social variables on SS-QoL scores. In this analysis, t-tests were applied to ascertain the variables' statistical significance, using the backward elimination method for entering variables in the model.

The adequacy of the adjustment was verified through application of Snedecor's F distribution test and by calculating the adjusted coefficient of determination (R^2^). The model's premises were evaluated using the Lilliefors test for normality in the residuals, and the Durbin-Watson test was used for verifying autocorrelation in the residuals. The level of significance of alpha was 0.05 for all analyses undertaken (p≤0.05).

## RESULTS

Regarding the sociodemographic and clinical characteristics of patients with stroke, patients were predominantly male (68.7%), married (63.4%) and retired (34.4%). A low percentage of patients were employed (10.7%) or had changed profession (2.3%) after IS. More than half of patients were older adults aged over 65 years old with a low family income and had attended school for up to four years. Regarding the stroke pattern, both cerebral hemispheres were equally affected. Lacunar syndrome was the most prevalent (35.9%), and the mean time of stroke after hospital discharge was eight months. On average, the time elapsed between the onset of stroke and obtaining treatment was 4 hours, and thrombolysis was performed in 18.3% of patients.

Regarding the risk factors for IS, we found a high prevalence of hypertension (85.5%). Furthermore, there was high prevalence of four other modifiable risk factors: sedentarism (80.9%), smoking (55%), alcoholism (48.1%) and dyslipidemia (47.3%).

Table 1 describes the clinical staging of IS. More than half of patients (53.4%) presented a high mRS score corresponding to moderate or severe disability (mRS 3-5). Additionally, over half of patients (54.2%) presented NIHSS scores reflecting moderate severity (5 to 15 points), and nearly half (42.7%) scored less than 20 points on the MMSE. Although this score was low, it may be related to the subjects' low level of education.

Table 2 presents the results of the SS-QoL scores. The median score for more than half of patients assessed was 151, indicating poor QoL. The most affected domains were as follows: Work/Productivity, Social Roles, Personality, Energy and Family Roles. Alternatively, the least affected domain was Vision.

Table 3 describes the association between QoL and the sociodemographic and clinical variables of stroke patients. The total SS-QoL score was lower for patients with TACS than for people with other syndromes (*p*<0.001). Compared with the group of patients who did not use an orthosis, the group of patients who used an orthosis presented a significantly lower score for QoL (*p*<0.001). Moreover, SS-QoL was significantly negatively correlated with mRS and NIHSS scores, indicating lower QoL among patients with worse functional status and higher IS severity scores (*p*<0.001).

Table 4 describes the multiple linear regression model obtained for the score for post-IS QoL. The QoL score decreased by 40 points among patients who used orthoses, 33 points among patients diagnosed with TACS, 16 points among patients whose left cerebral hemisphere was affected and an increase of three points for each month after the IS ictus. In the final model of regression analysis, orthosis and a total anterior circulation infarct subtype of stroke led to a more marked reduction in QoL.

## DISCUSSION

This article presents the sociodemographic and clinical characteristics, risk factors, and aspects related to the QoL of 131 patients with IS who were evaluated between six and 12 months after IS. The final linear regression revealed that among other aspects, the use of an orthosis and a TACS subtype of IS led to an important reduction in QoL. To our best knowledge, there is no previous study in our region. Thus, this study is relevant because it relates clinical and social aspects to the magnitude of stroke and its repercussion for QoL among the population of a region in the Brazilian Northeast.

Consistent with other studies, men were predominant among patients with IS 2,17. The patients' median age was 65 years of age or older, indicating that individuals of an economically active age are affected by stroke 18. Although these patients were in a transition phase from adulthood to senescence, they were independent in performing their daily living activities and social roles. Thus, having a stroke at this age had repercussions in their lives, with implications for the society in which they live.

Regarding educational level, the median number of years in education found in our sample was similar to that in other Brazilian studies. A large majority of the patients had studied up to 4 years 9,12. The median family income was US$800 per month for a family of three people. Low educational level is related to an increase in the incidence of IS, mainly when combined with socioeconomic and cultural factors and difficulty in accessing information. A large number of studies from developed and underdeveloped countries have described a direct association among knowledge of stroke, income and education 19,20. Low educational level also impairs adherence to treatment and maintenance of a healthy lifestyle 10. This situation is routinely encountered in developing countries 12.

Thrombolysis was present in approximately one-quarter of patients. In one Brazilian study undertaken in São Paulo, 17.7% of patients with IS were treated with intravenous thrombolysis 10. Thrombolysis significantly improves prognosis and functionality as it reduces the sequelae of stroke. A recent meta-analysis highlighted better post-stroke recovery in patients treated with intravenous thrombolysis 21.

Few studies have analyzed QoL in thrombolysed individuals. Thrombolysed patients presented satisfactory QoL, considering 60% as the cutoff point for the SS-QoL score. In a study conducted in Germany with 302 patients who received intravenous thrombolysis, a significant improvement in functionality was observed. However, depressive symptoms and impaired QoL were observed in 23% and 25% of stroke patients, respectively, after 3 months of intravenous thrombolysis. Despite good functional outcomes, patients may still have depression and poor QoL 22. Another study performed in Finland assessed 53 consecutive patients after 1 year of thrombolysis. The majority of patients achieved a good outcome measured with the SS-QoL, NIHSS and Barthel index 23. In the present study, it was not possible to make this association as the number of thrombolysed individuals was small. Another possible explanation is that the SS-QoL score may not be sufficiently sensitive to detect changes in patients 6 to 12 months after stroke. Additionally, another study did not find significant differences in QoL among patients with or without thrombolysis 24

In the literature, hypertension is the main established risk factor for stroke. Blood pressure represents the most important risk factor for stroke. Blood pressure treatment is also important for secondary prevention. In addition, compared with coronary patients, post-stroke patients presented greater persistence of the risk factors hypertension and smoking 25.

In our sample, one factor that needs attention is the high prevalence of sedentarism, the second greatest risk factor after hypertension. The need for lifestyle changes and encouragement of physical activity is very important, given that it is a modifiable risk factor. One recent study investigated the physical activity profile of patients with stroke compared with that of healthy subjects of the same age. The patients with stroke spent more time seated and less time engaging in physical activity than did other subjects of the same age 26.

The main influences on QoL in post-stroke patients have also been described in previous reports. Higher QoL is associated with greater independence in daily living and mobility, a higher education level, and better socioeconomic level and social support. Conversely, worse QoL is associated with anxiety, depression and fatigue 27,28.

Another study published in Turkey evaluated 80 geriatric stroke patients. Patients were assessed within the first week after stroke and reevaluated with 3 months. The QoL scores of 80 patients were lower than those of general population. Work/Productivity was the most affected subscale in the geriatric population, but Mobility, Self-Care and Social Roles were also important items. In contrast, in the group of younger patients, Upper Extremity Function, Work/Productivity, Energy and Self Care were the most affected subscale items 29. Multiple regression analysis was also performed. The most relevant predictor of QoL was the functional status during the assessment. Thus, improving physical function may help to provide better QoL for stroke patients. 29

In the present study, the domains of Work/Productivity, Social Roles, Personality, Energy and Family Roles were the most affected in the SS-QoL. This result is in line with previous studies in which the same dimensions were affected, with the addition of Mobility, Language, and Upper Extremity Function 30,31. All these domains are essential for functionality and when compromised, affect the ability to undertake activities and restrict participation in daily tasks, including personal, social or work-related tasks.

SS-QoL is a measure that assesses the influence of different aspects of the domains of health in a broad manner and provides important information on QoL. However, comparing studies that investigate QoL after stroke is difficult as they diverge regarding the period of evaluation after the ictus. In the present study, time after stroke was carefully evaluated to avoid these inconsistencies.

A previous study evaluated participants' mean SS-QoL score. The SS-QoL score of stroke survivors was reduced by -33.77, with each increase on the scale due to disability assessed by the mRS. In the same way, scoring 12 points or more on the NIHSS on admission was a significant predictor for worse QoL measured using the SS-QoL 3 months post stroke 2. Another study noted that functional status was the most important factor associated with the occurrence of depression and low QoL 3 to 6 months after stroke 22.

Our study revealed that the most frequent type of ischemia was LACS. This subtype was also associated with better functionality and QoL. Indeed, post-discharge complications were more frequent in patients with TACS in previous studies 3. Arm and hand function in patients with TACS was significantly more compromised than that in patients with LACS and patients with PACS 32.

Another study evaluated predictors of short-term improvement after IS. Patients who had the best recovery-related variables had the following characteristics: non-TACS IS, young, normal or low blood pressure and no language deficits 33.

QoL in patients with stroke has been investigated in various studies. However, comparison of these studies is difficult due to the use of different scales, as well as the heterogeneity of the patients evaluated. The majority of studies on QoL used scales with low specificity and sensitivity to stroke patients 29. Moreover, there is a paucity of studies in the literature assessing QoL and its association with the subtypes of IS. Our results indicated lower SS-QoL for patients with TACS than for those with the other syndromes.

Regarding the frequency of returning to work, one study indicated that sex, age, level of education and severity of stroke were determining factors. Few studies have described the return to work. Overall, people in managerial or administrative positions and the self-employed present a greater frequency of returning to work 34. An appropriately prescribed orthosis can improve gait performance and control abnormal kinematics resulting from deficits in coordination caused by stroke. However, in the present study, the use of an orthosis was related to reduction in QoL. A recent study evaluated gait training with an ankle-foot orthosis. The authors described improvement in walking speed and balance when the intervention was associated with physiotherapy combining repetitive facilitative exercises 35.

Nonetheless, few studies have addressed the motor performance of patients with stroke who use orthoses, making it difficult to generalize from these findings, especially given that the evidence on the efficacy of orthosis is limited 36. Our study also has some confounders. A low level of education, low family income and living in distant rural cities make rehabilitation difficult. In addition, accessibility for people with disabilities is poor in Brazil, which worsens physically disabled people's access to the health system. This issue is associated with difficulty in obtaining an orthosis within a reasonable period as well as accessing appropriate training. It is also important to highlight that QoL is not necessarily associated with satisfaction with an orthosis. Potentially, a patient may have functional gains for activities of daily living as a result of using an orthosis; additionally, patient QoL may be affected by the severity of the condition and not by the orthosis 6. Moreover, patients with worse QoL were diagnosed with more severe stroke and had more compromised functionality.

Our study also described worse QoL in patients with left hemispheric stroke. Individuals who have lesions in the left hemisphere often have language impairments. Language impairments are in turn related to lower functional levels and poorer cognitive function, resulting in these patients needing greater support to perform their daily living activities. Thus, left hemispheric stroke results in worse QoL 37.

This study has some limitations, such as the relatively small sample, which made it difficult to detect relevant differences between subgroups. Other aspects related to self-isolation, frustration and depression, as well as factors such as family support, social support and sleep disorders—which may also influence QoL—were not analyzed. Alternatively, this is one of the first local studies and one of the few Brazilian studies to assess the QoL of patients with IS. Moreover, we carefully evaluated clinical and sociodemographic factors that may be associated with QoL.

The present study described an inversely proportional relationship between the severity of stroke, disability and QoL. The IS subtype TACS, use of orthosis and higher NIHSS and Rankin scores were related to worse QoL. The early identification of these factors could promote better interventions for patients with IS, minimizing disabilities and improving QoL.

## AUTHOR CONTRIBUTIONS

Ramos-Lima MJ conceived the research, collected the data, interpreted the collected data, wrote the first draft of the manuscript and approved the final version of the manuscript. Brasileiro IC conceived the research, interpreted the collected data, reviewed the manuscript, collected data and approved the final version of the manuscript. De Lima TL interpreted the collected data, reviewed the manuscript and approved the final version of the manuscript. Braga-Neto P conceived the research, interpreted the collected data, reviewed the manuscript and approved the final version of the manuscript.

## Figures and Tables

**Table 1:** Clinical Staging of ischemic stroke patients.

Variables		n	%	CI 95%	
Modified Rankin Scale					
0 to 2 points		61	46.6	37.9	55.5
3 to 5 points		70	53.4	44.5	62.1
NIHSS on admission					
Very severe [>22 points]		13	9.9	5.6	16.7
Severe [16 to 22 points]		12	9.2	5.0	15.8
Moderate [5 to 15 points]		71	54.2	45.3	62.8
Minor [<5 points]		35	26.7	19.5	35.3

	Mean	SD	Median	IQR^a^	*p*-value^b^
Modified Rankin Scale	2.37	1.43	3.0	3.0	<0.001
NIHSS	10.34	7.17	9.0	11.0	<0.001
MMSE	19.09	6.84	20.0	10.0	<0.001

CI 95%, Confidence Interval; NIHSS, National Institutes of Health Stroke Scale; MMSE, Mini-Mental State Examination.

aIQR, interquartile range;^ b^ Lilliefors test.

**Table 2:** Score of the Stroke Specific Quality of Life Scale in patients with ischemic stroke.

Score by SS-QoL sub-domains	Mean	SD	Median	IQR^a^	*p-v*alue^b^
Energy (Total score=15)	8.56	4.82	8.0	11.0	<0.001
Family Roles (Total score=15)	8.56	4.20	8.0	8.0	<0.001
Language (Total score=25)	17.45	6.61	19.0	12.0	<0.001
Mobility (Total score=30)	18.75	8.38	20.0	14.0	<0.001
Mood (Total score=25)	16.55	7.10	18.0	13.0	<0.001
Personality (Total score=15)	8.24	4.49	7.0	9.0	<0.001
Self Care (Total score=25)	18.00	7.46	22.0	14.0	<0.001
Social Roles (Total score=25)	11.71	7.02	10.0	12.0	<0.001
Thinking (Total score=15)	9.26	3.95	9.0	7.0	<0.001
Upper Extremity Function (Total score=25)	16.30	7.90	18.0	16.0	<0.001
Vision (Total score=15)	12.19	3.43	14.0	5.0	<0.001
Work/Productivity (Total score=15)	7.66	4.78	6.0	9.0	<0.001
SS-QoL (Total score=245)	153.24	48.95	151.0	77.0	0.005

^a^IQR, interquartile range; ^b^ Lilliefors test.

**Table 3:** Association between the characteristics of patients with ischemic stroke and quality of life.

Variables	n	Mean ranks	Test statistics	*p-v*alue
Sex				
Male	90	69.28	1550.01	0.1431
Female	41	58.80		
Bamford Classification*				
Total Anterior Circulation Syndrome^a^	36	35.58	32.122	<0.0012
Partial Anterior Circulation Syndrome^b^	19	79.97		
Lacunar Syndrome^b^	47	78.20		
Posterior Circulation Syndrome^b^	29	74.83		
Use of ortheses				
No	72	83.77	844.51	<0.0011
Yes	59	44.31		
Rehabilitation after stroke				
No	59	66.96	2067.51	0.7941
Yes	72	65.22		
Age [years]			-0.0243	0.788
Income			0.0213	0.808
Educational level			0.0143	0.872
Time post stroke			0.1293	0.141
Modified Rankin Scale			-0.6843	<0.001
NIHSS Scale			-0.5103	<0.001

1Mann-Whitney U test; 2Kruskal-Wallis H Test; 3Spearman's rank correlation coefficient. *Different letters in superscript (a, b, c, d) correspond to the means of different ranks using Dunn's test at the level of 5%. NIHSS, National Institutes of Health Stroke Scale.

**Table 4:** Multiple linear regression model for quality of life of patients with stroke based on the Stroke Specific Quality of Life Scale.

Variables	Coef.	Standard error	t-test	*p*-value
Intercept	163.02	15.22	10.71	<0.001
Orthosis (yes)	-40.68	7.48	-5.43	<0.001
BAMFORD (Lacunar Syndrome)				
Partial Anterior Circulation Syndrome	0.005	10.62	0.00	0.999
Total Anterior Circulation Syndrome	-33.64	9.45	-3.56	<0.001
Posterior Circulation Syndrome	1.14	9.26	0.12	0.902
Cerebral hemisphere (right)				
Left	-16.99	7.36	-2.31	0.023
Both	-18.26	23.01	-0.79	0.429
Time after stroke (months)	3.30	1.80	1.84	0.068
Goodness of fit				
F Test=12.31	gl1: 7		gl2:123	<0.001
Lilliefors test for normality of residuals:			W=0.98	0.267
Durbin-Watson test for autocorrelation in the residuals			DW=1.64	0.019
Adjusted R^2^:	0.379			

df: degree of freedom. W: Lilliefors test for normality. DW: statistic Durbin-Watsu.
